# A new radiological measurement method used to evaluate the modified transconjunctival orbital fat decompression surgery

**DOI:** 10.1186/s12886-021-01911-9

**Published:** 2021-04-13

**Authors:** Bei Li, Li Feng, Huamin Tang, Liuzhi Zeng, Wei Lin

**Affiliations:** 1 Department of Ophthalmology, Chengdu First People’s Hospital, No.18 Wanxiang North Road, Chengdu, 610041 Sichuan Province China; 2 Department of Radiology, Chengdu First People’s Hospital, Chengdu, Sichuan Province China

**Keywords:** Radiological, Fat decompression, Thyroid-associated ophthalmopathy

## Abstract

**Purpose:**

A new radiological method was used to evaluate the plastic effect of modified transconjunctival orbital fat decompression surgery in patients with inactive thyroid-associated ophthalmopathy.

**Methods:**

In this study, 10 inactive patients (14 eyes) with moderate to severe thyroid-associated ophthalmopathy were selected. The patients underwent modified transconjunctival orbital fat decompression surgery. According to the results of a spiral CT scan before and 6 months after the surgery, the INFINITT system workstation was used to measure the eyeball protrusion value. According to the results obtained by the PHLIPS IntelliSpace Portal elliptical area and line segment measurement tools, the standard elliptical vertebral volume formula was used to calculate the muscular cone inner volume. Changes in eyeball protrusion and the inner volume of the muscular cone before and after surgery were examined. Statistical analysis of the correlation between the two parameters was performed.

**Results:**

Radiological measurement results confirmed that removing the orbital fat in the muscle cone during surgery was effective for alleviating eyeball protrusion in patients with thyroid-associated ophthalmopathy (*P* < 0.05). This surgery caused an obvious change in the muscle cone inner volume (*P* < 0.05). And there was significant correlation between changes in eyeball protrusion and muscle cone inner volume (r = 0.797, *P* = 0.0006, *P* < 0.05).

**Conclusion:**

The radiological assessment method used in this study is simple and easy to implement. For inactive patients with moderate to severe thyroid-associated ophthalmopathy who just want to improve their appearance, the modified orbital fat decompression surgery is worth considering.

## Background

Thyroid-associated ophthalmopathy (TAO), also known as Graves’ ophthalmopathy (GO) or thyroid eye disease (TED), is an autoimmune disease that accounts for the highest incidence of orbital diseases in adults. In March 2016, the European Thyroid Association/European Group on Graves’ Orbitopathy (EUGOGO) published guidelines for the management of GO. Based on the guidelines, surgery is an effective treatment for moderate to severe inactive patients, patients with active severe exposed ocular surface inflammation, and active patients with oppressive optic neuropathy [1]. However, more inactive patients require surgery because of their appearance [2]. A study had shown that orbital fat decompression surgery can significantly improve the overall quality of life with a low complication rate [3].

In most of the literature, during orbital fat decompression surgery, not only the orbital fat inside the muscle cone, but also the orbital fat outside the muscle cone is removed [4–7]. However, based on the anatomy of the orbit, most of the orbital fat outside the muscle cone is located in the front of the orbit, and the most direct force that causes exophthalmos comes from the fat in the muscle cone behind the eyeball. In our experience, removing the orbital fat outside the muscle cone had no effect on improving the exophthalmos and might affect the position of the eyeball. Some surgeons only removed the fat behind the ball during the surgery without the need to disturb the anterior orbital fat pads, and achieved obvious surgical effect [2].

In order to explore the relationship between changes in eyeball protrusion and changes in cone volume, we selected moderate to severe inactive patients who underwent the modified transconjunctival orbital fat decompression surgery at the department of ophthalmology in Chengdu First People’s Hospital. We conducted a retrospective study using the results of preoperative and postoperative computed tomography (CT) examinations. During the study we used a new radiological measurement method to acquire data.

## Materials and methods

### Case source and inclusion criteria

This study was approved by the Ethics Committee of Chengdu First People’s Hospital (certificate approval number:2021-WZ-001). The study adhered to the tenets of the Declaration of Helsinki. The patients’ partial appearance photos and orbital CT scan results in this article had been approved by the patients. The written informed consent—for both study participation and publication of identifying information/images in an online open-access publication was obtained from the patients. A copy of the written informed consent is available for review by the Editor of this journal.

The cases included 14 eyes of 10 TAO patients (mean age: 41.8 ± 9.7 years; sex: 5 males and 5 females; mean course of disease: 22.4 ± 7.2 months) who underwent orbital fat decompression surgery in 2019. Case inclusion criteria: all patients met the criteria for moderate to severe patients in the inactive phase based on the guidelines formulated by EUGOGO [1], and their thyroid function and exophthalmos had been stable for more than half a year. Case exclusion criteria: patients had suffered from other orbital diseases or received any previous orbital surgery, and according to the results of the preoperative CT scan, the extraocular muscles were thickened significantly.

### Surgical technique

**Fig. 1:**
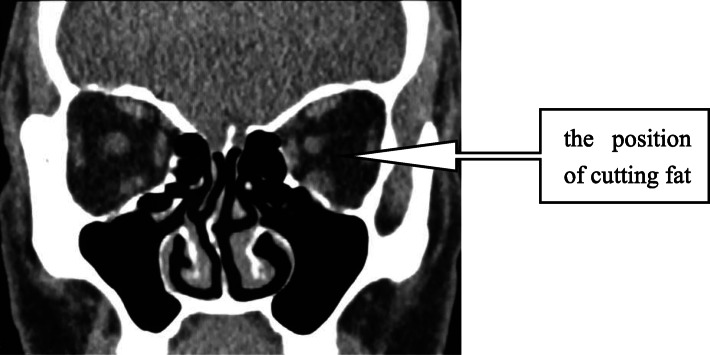
The position of cutting fat. It’s in the muscle cone and between the inferior rectus and the external rectus

Based on the surgical technique for deep orbital fat resection [8] from *“Chinese Ophthalmology”*, first, we axially cut the lateral canthus with surgical scissors, cut off the lower branch of the lateral canthal ligament, and freed the lower eyelid. Next, we cut the bulbar conjunctiva along the inferior fornix and entered the surgical gap between the orbital periosteum and extraocular muscles. Then, we entered the muscle cone from the gap between the inferior rectus muscle and the lateral rectus muscle by incising the intermuscular septum, and removed the fat tissue in the muscle cone carefully with hemostatic forceps. However, we did not remove the orbital fat outside the muscle cone. Finally, the bulbar conjunctiva and lateral canthus were sutured (Fig. 1). All surgeries were performed by Dr. Bei Li.

### Method of spiral CT scanning

All patients underwent non-enhanced spiral CT scans (PHILIPS, Netherlands) before and 6 months after surgery at the department of radiology in Chengdu First People’s Hospital. Each patient was scanned in a supine position with both eyes fixed on a target. The Frankfurt line was used as the baseline, and a spiral volume scan was performed (scan parameters: layer thickness 1 mm, layer spacing 1 mm, pitch 0.6, matrix 512 × 512, window width 300 HU and window position 35 HU). The obtained images were all transmitted to the picture archiving and communication systems (PACS, PHLIPS, Netherlands) for the measurement of eyeball protrusion and muscle cone inner volume.

### Method for measuring eyeball protrusion

**Fig. 2:**
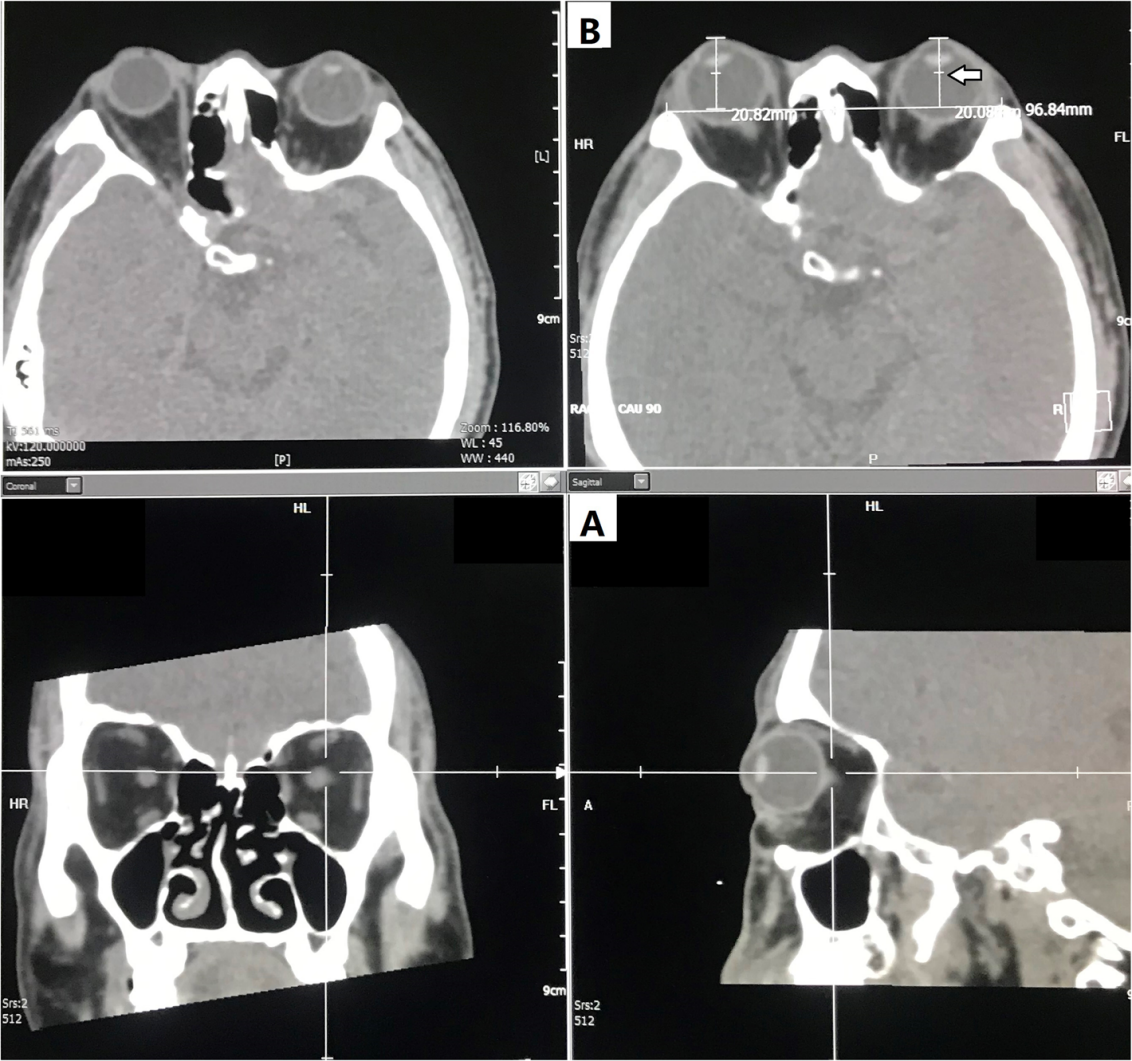
Method for measuring eyeball protrusion. The length of the line segment pointed by the arrow is the value of eyeball protrusion

Multi-planner reformation (MPR) was performed on the spiral CT image data, and axial, sagittal and coronal images of the orbit were obtained simultaneously (Fig. 2). Using the sagittal image as the benchmark (Fig. 2A), we determined the most prominent layer of the eyeball and used the corresponding axial image as the standard layer to measure eyeball protrusion (Fig. 2B). On the standard level of the axial position, we connected the left and right highest points of the outer edge of the orbits as a reference line, passed the furthest point of the front projection of the eyeball, drew a perpendicular to the reference line, and then measured the distance of the perpendicular as eyeball protrusion value. Three skilled radiologists (Li Feng, Huamin Tang and Wei Lin) independently completed the measurement of each data and obtained the mean. They were masked and double blind.

### Method for measuring the muscle cone inner volume (Fig. 3)

#### Method for determining the standard measurement position

**Fig. 3:**
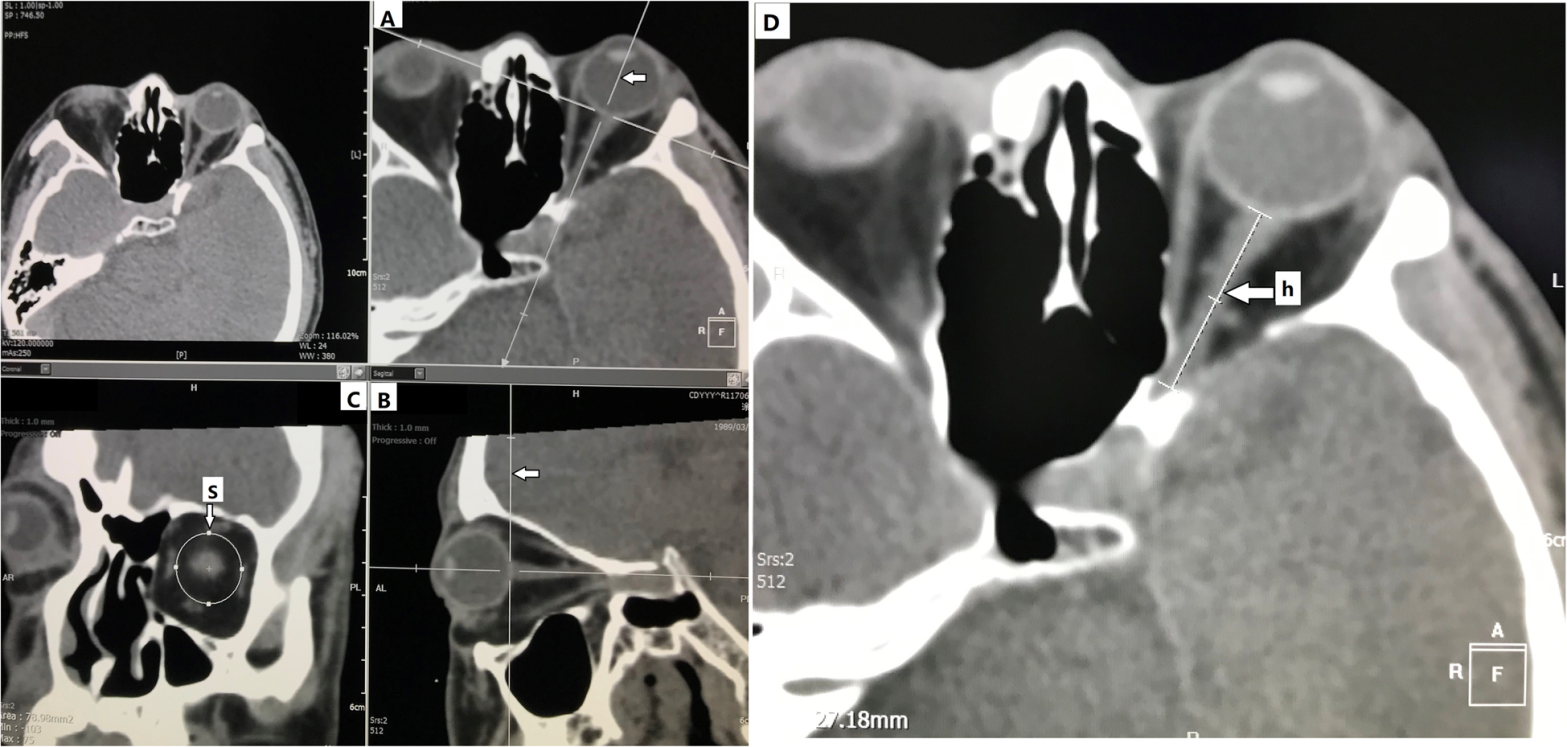
Method for measuring the muscle cone inner volume. On the **a** the line pointed to by the arrow is the sagittal axis. On the **b** the line pointed to by the arrow is the coronal axis. On the **c** the area of the ellipse pointed to by the arrow is the muscle cone bottom area (S), and the length of the line segment pointed to by the arrow is the muscle cone height (h)

We used the “tangential coronal position” as the standard measurement position. First, on the axial image of the orbit with the optic nerve displayed (Fig. 3A), we adjusted the sagittal axis to overlap with the optic nerve long axis to obtain the reference sagittal position (Fig. 3B). Second, on the reference sagittal image, we adjusted the coronal axis to be tangential to the position where the optic nerve exited the eyeball to obtain the “tangential coronal position” for measurement (Fig. 3C).

#### Method for calculating the muscle cone inner volume

Because the muscle cone anatomy was similar to an elliptical cone, we used the standard elliptical vertebral body volume formula (V=Sh/3, V: vertebral body volume, S: cone base area, h: vertebral height) to calculate the muscle cone inner volume.

First, on the “tangential coronal position”, using the elliptical area measurement tool, with the optic nerve as the measurement center, we manually outlined and adjusted the elliptical edge to be tangential to the inner edges of the four extraocular muscles. The resulting elliptical area was the muscle cone bottom area (S) (Fig. 3C). Then, on the reference transection with the optic nerve displayed, we used the line measurement tool to measure the distance from the beginning of the intraorbital segment of the optic nerve to the beginning of the optic canal. The resulting distance was the muscle cone height (h) (Fig. 3D). Three skilled radiologists (Li Feng, Huamin Tang and Wei Lin) independently completed the measurement of each data and obtained the mean. They were masked and double blind.

### Statistical methods

GraphPad Prism 9.0.0 (USA, GraphPad Software) was used for statistical analysis. Based on the results of a normality distribution test, all data were normally distributed. Paired t-tests and Pearson product-moment correlation were used. Paired t-tests was used to compare the changes of eyeball protrusion and muscle cone inner volume between preoperative and postoperative. Pearson product-moment correlation was used to analyze the correlation between the two changes. The correlation coefficient was represented byr. The results were considered statistically significant if *P* < 0.05.

## Results

### Demographic and clinical characteristics of studied patients (Table 1)

**Table 1:** Demographic and clinical characteristics of studied patients

Case	Eyes	Age(years)	Gender	Course of disease (months)	Steroid pulse	CAS score	Optic neuropathy	Diplopia
1	right	28	female	14	yes	1	no	no
2	right	38	male	18	yes	0	no	no
3	left	39	female	20	yes	0	no	no
4	both	50	male	16	yes	0	no	no
5	both	55	female	30	yes	1	no	no
6	left	30	female	16	no	0	no	no
7	both	35	female	22	yes	0	no	no
8	right	45	male	22	yes	0	no	no
9	left	56	male	35	no	1	no	no
10	both	42	male	31	yes	0	no	no

### Preoperative and postoperative appearance photos of a subset of patients (Fig. 4)

**Fig. 4:**
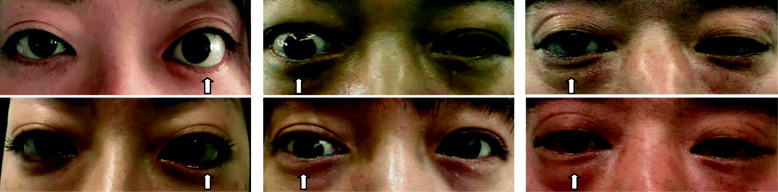
Preoperative and postoperative appearance photos of a subset of patients. The arrows point to the surgical eyes

### Results of eyeball protrusion measurements (Table 2, Fig. 5)

**Table 2:** Preoperative and postoperative eyeball protrusion ($\overline{x}$ ± *s*, *n* = 3)

Eyes	Preoperative protrusion (mm)	Postoperative protrusion (mm)
1	20.82 ± 0.35	18.26 ± 0.31
2	20.08 ± 0.16	16.32 ± 0.15
3	21.77 ± 0.26	17.94 ± 0.17
4	20.46 ± 0.24	17.02 ± 0.05
5	18.68 ± 0.36	15.31 ± 0.21
6	21.58 ± 0.12	18.87 ± 0.25
7	23.36 ± 0.31	20.41 ± 0.16
8	23.08 ± 0.11	20.27 ± 0.19
9	19.76 ± 0.32	16.84 ± 0.33
10	18.32 ± 0.25	16.13 ± 0.28
11	19.85 ± 0.22	17.15 ± 0.23
12	19.81 ± 0.30	16.85 ± 0.32
13	20.31 ± 0.16	16.99 ± 0.22
14	19.59 ± 0.27	16.55 ± 0.09

*P* < 0.05 Preoperative protrusion vs Postoperative protrusion (paired t - test)

**Fig. 5:**
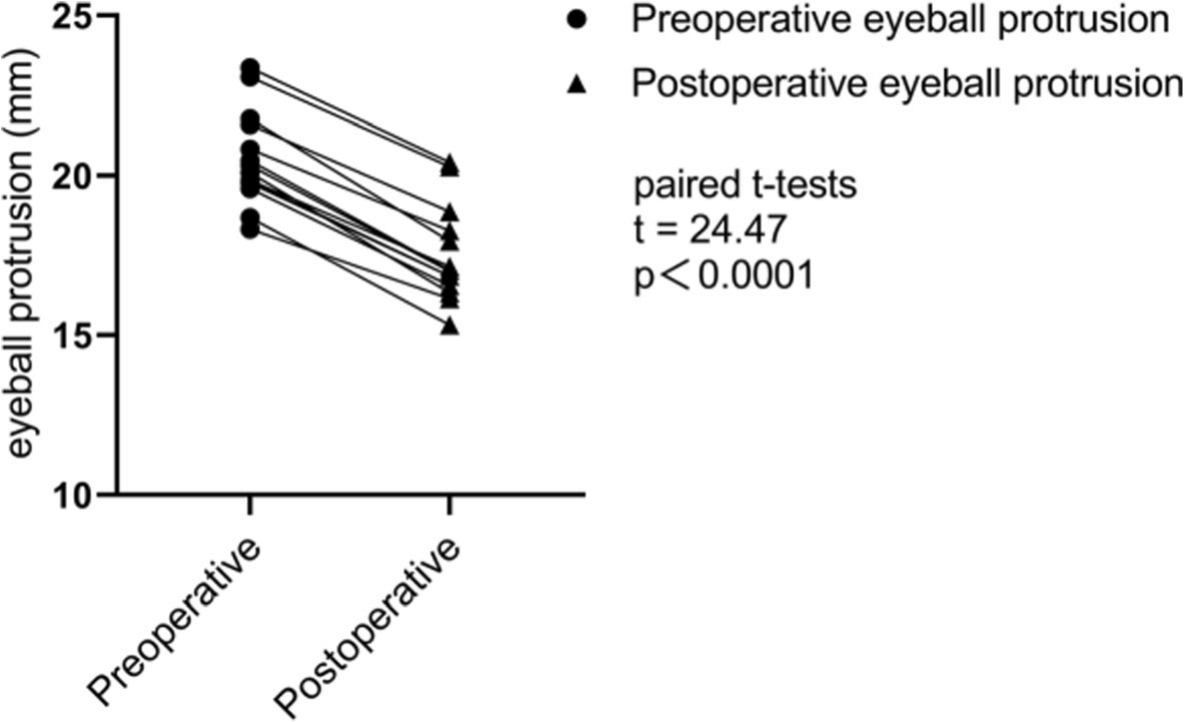
Preoperative protrusion vs Postoperative protrusion, t = 24.47, *P*<0.0001. (paired t- tests)

### Results of muscle cone inner volume measurements (Table 3, Fig. 6)

**Table 3:** Preoperative and postoperative muscle cone inner volume ($\overline{x}$ ± *s*, *n* = 3)

Eyes	Preoperative volume (mm^3^)	Postoperative volume (mm^3^)
1	6258.95 ± 55.11	4407.76 ± 63.28
2	6222.78 ± 80.14	4019.14 ± 35.22
3	7609.94 ± 39.26	5685.99 ± 41.65
4	7321.41 ± 40.35	5425.61 ± 65.15
5	6377.56 ± 33.16	4653.23 ± 77.01
6	7839.59 ± 52.51	6275.37 ± 56.23
7	8192.59 ± 48.67	6512.58 ± 64.27
8	7558.99 ± 32.07	6149.72 ± 49.37
9	6044.08 ± 45.80	4451.24 ± 50.03
10	5474.37 ± 81.25	4458.18 ± 36.25
11	6251.20 ± 29.78	4717.31 ± 46.18
12	5997.75 ± 57.20	4182.40 ± 67.74
13	7398.49 ± 42.25	5484.63 ± 58.45
14	7422.71 ± 63.31	5519.61 ± 85.31

*P* < 0.05 Preoperative protrusion vs Postoperative volume (paired t - test)

**Fig. 6:**
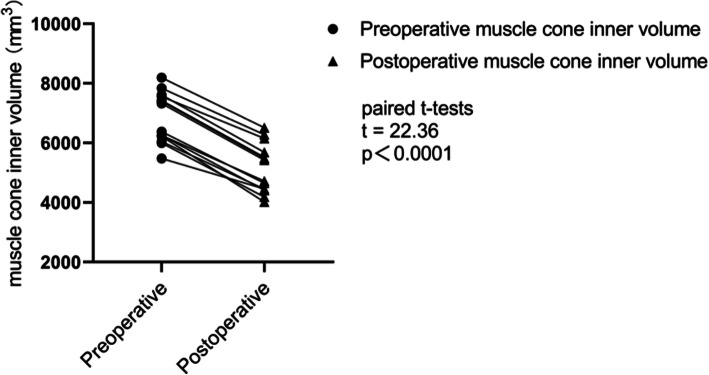
Preoperative muscle cone inner volume vs Postoperative muscle cone inner volume, t = 22.36, *P*<0.0001. (paired t- tests)

### Correlation analysis results of differences in protrusion and volume (Table 4, Fig. 7)

**Table 4:** Preoperative and postoperative differences in protrusion and in volume ($\overline{x}$ ± *s*, *n* = 3)

Eyes	Difference in protrusion (mm)	Difference in volume (mm3)
1	2.56 ± 0.22	1851.19 ± 50.25
2	3.76 ± 0.14	2203.64 ± 67.13
3	3.83 ± 0.16	1923.95 ± 48.27
4	3.44 ± 0.34	1895.80 ± 72.15
5	3.37 ± 0.26	1724.33 ± 88.06
6	2.71 ± 0.19	1564.22 ± 35.15
7	2.95 ± 0.14	1680.01 ± 64.24
8	2.81 ± 0.07	1409.27 ± 79.29
9	2.92 ± 0.30	1592.84 ± 57.13
10	2.19 ± 0.27	1016.19 ± 54.23
11	2.70 ± 0.32	1533.89 ± 93.20
12	2.96 ± 0.20	1815.35 ± 84.15
13	3.32 ± 0.26	1913.86 ± 80.18
14	3.04 ± 0.11	1903.10 ± 41.21

*r* = 0.797, *P* = 0.0006 Difference in protrusion vs Difference in volume (Pearson product-moment correlation)

**Fig. 7:**
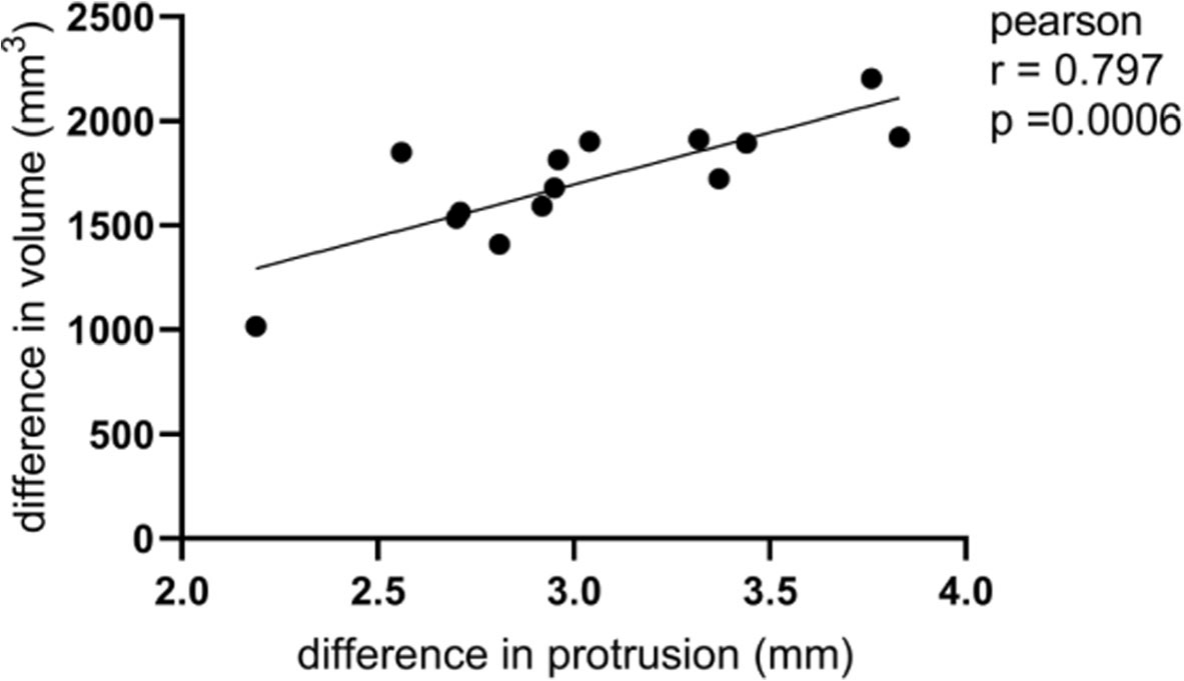
Difference in protrusion vs Difference in volume, r = 0.797, *P* = 0.0006. (Pearson product-moment correlation)

## Discussion

In recent years of research, some scholars measured the eyeball protrusion of TAO patients on the images obtained by CT scan, and they determined the most forward measurement position of the eyeballs by the the thickest position of the lens [9, 10]. For TAO patients without myopathy, the CT slice of the thickest lens corresponds to the apex of the cornea, which is the most protruding position of the eyeball. However, since the apex of the cornea and interzygomatic line cannot be included in the same plane on a two-dimensional (2D) CT scan, the level of the measured CT slice may not correspond to the area of maximal proptosis [11]. For TAO patients with varying degrees of restrictive strabismus, the apex of the cornea corresponding to the CT slice may not be the most protruding position of the eyeball. Therefore, the method of determining the measurement position of the eyeball protrusion through the thickest position of the lens is limited. In our study, we used the most prominent position of eyeballs in the sagittal position to determine the corresponding axial position for measurement. The method is more intuitive and easy. And it can avoid the measurement difficulty and error caused by the deviation of the eyeball position.

The fascial tissue between the extraocular muscles can’t be clearly displayed on the images obtained by CT. So there is no software can directly measure the inner volume of the muscle cone. In the previous literature, the use of software can only measure the retrobulbar orbital volume [7]. In our study, to measure the muscle cone inner volume, we simulated the muscle cone as an elliptical cone. In the “tangential coronal position”, we used measurement tools of the system workstation to obtain the required data and calculate the muscle cone inner volume. This method is effective and feasible.

The muscular cone is an independent and relatively closed structure surrounded by extraocular muscles and fascia in the orbit. An increase and decrease in intraconal fat mass will most directly affect the increase and decrease in eyeball protrusion. Most of the extracone fat is present in the front of the orbit and around the eyeball. So the extracone fat does not directly lead to the protrusion of the eyeball, and has a certain effect on stabilizing the position of the eyeball. Therefore, in our study, we only removed the fat inside the muscle cone and retained the fat outside the muscle cone. After the modified surgery, the patients got satisfactory appearance. The study results confirmed that only removing the orbital fat in the muscle cone during surgery had a significant effect on the eyeball protrusion and muscle inner volume of TAO patients (*P* < 0.05). And there was significant correlation between changes in eyeball protrusion and muscle cone inner volume (r = 0.797, *P* = 0.0006, *P* < 0.05).

At present, the main surgical methods used to relieve eyeball protrusion are bony orbital decompression surgery, orbital fat decompression surgery, and a combination of the two methods. The main surgical approaches include the transcutaneous approach, transconjunctival approach, and transnasal approach. If the patient does not have severe ocular surface inflammation or oppressive optic neuropathy and the purpose of surgery is only to improve their appearance, the removal of orbital fat in the muscle cone is a surgical method worth considering. This surgical method avoids damage to the normal orbital bone in bony orbital decompression surgery and avoids damage to the normal mucosal tissue of the nose in transnasal orbital decompression surgery. The radiological measurement method used in our research can effectively evaluate the effect of this surgery.

## Data Availability

The datasets during and/or analysed during the current study available from the corresponding author on reasonable request.
